# Prognostic Value of Neutrophil-to-Lymphocyte Ratio in Cats With Hypertrophic Cardiomyopathy

**DOI:** 10.3389/fvets.2022.813524

**Published:** 2022-03-14

**Authors:** Ryan C. Fries, Saki Kadotani, Jonathan P. Stack, Leah Kruckman, Gabrielle Wallace

**Affiliations:** ^1^Department of Veterinary Clinical Medicine, College of Veterinary Medicine, University of Illinois at Urbana-Champaign, Urbana, IL, United States; ^2^VCA Loomis Basin Veterinary Clinic, Loomis, CA, United States; ^3^Pacific Northwest Pet ER and Specialty Center, Vancouver, WA, United States

**Keywords:** mortality, inflammation, neutrophil-to-lymphocyte ratio, prognosis, hypertrophic

## Abstract

**Objective:**

To assess the prognostic value of neutrophil-to-lymphocyte ratio (NLR) for cardiac death in cats with hypertrophic cardiomyopathy.

**Study Design:**

Prospective observation study.

**Animals:**

Ninety-six client-owned cats.

**Methods:**

Complete blood count samples were collected from 38 healthy and 58 cats with hypertrophic cardiomyopathy (HCM), and the NLR ratios were analyzed. All cats had echocardiographic measurements performed on the same day as blood collection. Spearman rank correlation was used to assess the relationship between echocardiographic measurements and NLR. Long-term outcome data were obtained, and time to cardiac death and variables associated with cardiac death were analyzed using Kaplan–Meier survival curves and Cox proportional hazards models, respectively.

**Results:**

The NLR was significantly higher in cats with confirmed congestive heart failure. When evaluating HCM patients, cats in the third NLR tertile had a significantly higher risk of cardiac death with a hazard ratio of 10.26 (95% CI: 1.84–57.14; *p* = 0.0001) when compared with that of patients in the first tertile. NLR was significantly associated with echocardiographic measures of left atrial size, left auricular function, the presence of left atrial spontaneous echo contrast (SEC), and thrombus formation.

**Conclusions and Clinical Relevance:**

Increased NLR is a negative prognostic indicator in cats with HCM.

## Introduction

Hypertrophic cardiomyopathy (HCM) is the most common acquired cardiac disease in cats, affecting ~16–34% of apparently healthy cats (1, 2).The prognosis for cats with HCM is highly variable, with reported median survival times between 2 and 4, 418 days (3–6). Risk stratification and prognosis in feline HCM is based largely on echocardiographic indices of left atrial size and left ventricular function and wall thickness (7). While echocardiography is excellent at evaluating heart size and function, it requires advanced technical expertise and is not widely available to most general practitioners. Several biomarkers previously identified in humans have been successfully translated into veterinary species, including N-terminal probrain natriuretic peptide (NT-proBNP), cardiac troponin I (cTnI), and cardiac troponin T (cTnT) (8–13). These biomarkers have been found to be useful to identify patients who should undergo further cardiac evaluation, and severely elevated cTnI is a predictor of adverse cardiac outcomes in cats with HCM (10). Another study evaluated the association of NT-proBNP in cats with heart failure and found that cats with a larger percentage decrease in NT-proBNP during hospitalization had longer survival times (14). Unfortunately, absolute NT-proBNP concentrations were not associated with survival, and three precise time points were required to monitor the changes in NT-proBNP, resulting in onerous guidelines for veterinarians and extensive costs for owners. In exploring new approaches to determe prognosis in cats with HCM, we evaluated a widely available hematological test, the neutrophil-to-lymphocyte ratio (NLR).

The NLR is an easily accessible and widely available hematological marker of inflammation and stress (15–20).It also serves as a good prognostic marker and has been studied in veterinary patients with oncological and systemic inflammatory disorders. In humans, previous research has revealed a predictive value of NLR in peripheral artery disease, calcific aortic stenosis, coronary artery disease, heart failure, pulmonary hypertension, and recently HCM (21–23). A higher NLR is associated with poor prognosis in most studies. To date, the NLR has not been evaluated in cats with HCM.

The aim of this study was to determine the prognostic value of NLR in cats with HCM. We hypothesized that an elevated NLR would be significantly associated with reduced time to cardiac death.

## Materials and Methods

### Animals

Ninety-six client-owned cats were prospectively recruited and enrolled over a 5-year period from April 2016 to May 2021. Tests performed included physical examination, indirect blood pressure by Doppler method, CBC, biochemical analysis including total thyroxine concentration, urinalysis, transthoracic echocardiogram, and thoracic radiographs. For inclusion, cats had to have all diagnostic tests performed and blood samples collected within 24-h of presentation to the hospital. Exclusion criteria included cats with congenital or non-HCM cardiac disease, systemic hypertension (systolic blood pressure > 160 mmHg), pulmonary hypertension estimated by Doppler echocardiography (systolic pulmonary artery pressure > 55 mmHg), corticosteroid use in the previous 3 months, hyperthyroidism, or any systemic illness that may limit survival significantly (i.e., neoplasia). All patients with heart failure had to be receiving furosemide orally upon discharge from the hospital.

Cats were considered healthy if all diagnostic tests were within normal limits. A diagnosis of HCM was determined by a diastolic left ventricular free-wall or interventricular septal thickness ≥6 mm on echocardiography (24). Heart failure was determined by thoracic radiographs showing cardiomegaly, vertebral heart score >8.1 on the lateral projection, as well as evidence of either pulmonary edema or pleural effusion with concurrent clinical signs such as tachypnea (respiratory rate >50/min in the examination room or resting respiratory rate >40/min at home, or both) and dyspnea that must resolve with diuretic treatment. Cats were staged as healthy, subclinical (Stage B), or heart failure (Stage C) according to the ACVIM consensus statement guidelines for classification, diagnosis, and management of cardiomyopathy in cats (25). None of the healthy cats or Stage B cats with HCM received medications other than topical heartworm and flea prevention at the time of enrollment.

### Clinical Data

#### Echocardiogram

All cats had a complete transthoracic echocardiographic examination performed by a board-certified cardiologist or cardiology resident under direct supervision, by use of recommended standardized transthoracic imaging planes (26–28). A diagnosis of HCM was determined by a diastolic left ventricular free-wall or interventricular septal thickness ≥6 mm on echocardiography (24). Echocardiographic measurements obtained for each cat included, left ventricular (LV) size and function performed using standard right parasternal short-axis and long-axis views and left apical parasternal long-axis views. Two-dimensional variables measured included LV internal dimensions at end-diastole (LVIDd) and end-systole (LVIDs), LV free-wall thickness at end-diastole (LVFWd) and end-systole (LVFWs), and interventricular septal thickness at end-diastole (IVSd) and end-systole (IVSs). The LV fractional shortening was calculated using the following formula: LV FS = [LVIDd – LVIDs]/LVIDd × 100%. Assessment of left atrial (LA) size was performed from standard right parasternal long-axis and short-axis views. Variables measured included LA diameter (LA_SAX_) and aortic diameter (Ao) measured from a right parasternal short-axis view in early diastole timed to the earliest frame in which the closed aortic value cusps could be visualized. The ratio between LA_SAX_ and Ao (LA:Ao) were calculated. Additionally, the LA septal-to-free wall dimension maximum (LAD_Max_) and minimum (LAD_Min_) were measured from the right parasternal long-axis four-chamber view. The LAD_Max_ and LAD_Min_ were measured mid-chamber approximately parallel to the mitral annuls at end LV systole immediately prior to mitral valve opening (LAD_Max_) and end LV diastole immediately after mitral valve closure (LAD_Min_). The LA fractional shortening was calculated using the following formula: LA FS = [LAD_Max_ – LAD_Min_]/LAD_Max_ × 100%. Left atrial appendage emptying velocities (LAA) were recorded using pulsed-wave Doppler from the left cranial short-axis view, and the presence or absence of spontaneous echo contrast (SEC) or thrombus was noted. Transmitral velocities were recorded using pulsed-wave Doppler from a left apical parasternal long-axis view with the sampling gate place in line with color Doppler flow at the level of the open mitral valve tips. Variables measured included peak velocity of early diastolic transmitral flow (E), deceleration time of early diastolic transmitral flow (DT_E_), and peak velocity of late diastolic transmitral flow (A). Ratio between peak E to peak A (E:A) was calculated. Tissue Doppler imaging (TDI) was performed with the highest available transducer frequency to record the velocity of lateral mitral annular motion from the left apical parasternal long-axis view with the sampling gate placed on the lateral mitral annulus. The following variables were measured: peak early diastolic velocity (E′), peak late diastolic velocity (A′), and peak systolic velocity (S′).

#### Health Panel

Whole blood was drawn from a peripheral vein within 24-h of presentation, and a complete blood count, biochemistry analysis, and total thyroid (T4) were assessed. The complete blood count was performed using an automated analyzer (Abbott Cell-Dyn 3700) and manually verified by a board-certified veterinary clinical pathologist.

#### Thoracic Radiographs

Thoracic radiography was performed at presentation for all cats with suspected heart failure or an enlarged left atrium on echocardiography using right lateral and ventrodorsal or dorsoventral projections. The presence and severity of pulmonary edema (none, mild interstitial density, moderate interstitial density, alveolar pattern, and severe consolidation) were assessed as well as the presence and severity of pleural effusion (none, mild, moderate, severe).

### Outcome

The outcome of the cats was determined by follow-up examinations and telephone interviews with the owner. The primary endpoint was sudden cardiac death or euthanasia for HF. When a cat died spontaneously or was euthanized, the investigator determined whether the cause of death was cardiac or noncardiac. In cases in which the cause of death was considered noncardiac, the reason for death or euthanasia was noted. Survival time was defined as the time from enrollment into the trial to the endpoint.

### Statistical Analysis

Statistical analyses were performed using commercially available software (GaphPad Prism, version 8, GraphPad Software, Inc, San Diego, CA, USA; MedCal for Windows, version 19.7, Ostend, Belgium). Normality was assessed using the Shapiro–Wilk test and continuous variables were expressed as mean ± standard deviation (SD) when normally distributed, or median and range when the distribution was nonnormal. Based on the results of the normality, comparisons between groups were assessed using one-way ANOVA or a Kruskal–Wallis test, and Spearman rank correlation was used to assess the relationship between echocardiographic measurements and NLR. Survival analysis was performed in cats that reached the primary endpoint (cardiac death) and all cats alive or dying of noncardiac disease were right-censored. The continuous variables, LAmax and NLR subclassified by tertile, were analyzed using Kaplan–Meier curves. To investigate independent prognostic factors, we used Cox proportional hazard analyses on all continuous variables. After the univariate analysis, only variables with *p*-value <0.1 were used in the multivariate analysis. The results were described as hazard ratios (HR) and 95% CI. Receiver operating characteristic (ROC) analysis was performed to assess the diagnostic accuracy of NLR to predict cardiac death. A diagnostic cutoff was chosen on the basis of the highest of various combinations of sensitivity and specificity using Youden's index (Y = sensitivity + specificity-1). *p*-value <0.05 was defined as statistically significant.

## Results

One-hundred and forty-four cats were eligible for inclusion after initial screening. Forty-eight were excluded because of concurrent non-HCM cardiac disease (mitral valve dysplasia 10, transient myocardial thickening 7, ventricular septal defect 4, atrial septal defect 2), hyperthyroidism (9), incomplete diagnostics (8), and systemic hypertension (8), leaving a total of 96 cats that met all inclusion criteria. Demographic, physical examination, echocardiographic, and hematologic data are summarized in Table 1.

**Table 1:** Demographic, physical examination, echocardiographic, and hematologic data in 96 cats.

	**Healthy**	**Stage B HCM**	**Stage C HCM**	**P-value**
Number of cats	38	29	29	
Age (years)	6.2 ± 1.2	6.7 ±0.75	7.5 ± 2.0	0.122
Follow up (days)	774 (45–1,500)a	456 (67–1,043)a	188 (19–546)b	<0.0001
Sex m/f (number)	24/14a	16/13b	18/11a	0.031
Weight (kg)	4.5 (3.1–5.5)	4.37 (3.3–5.6)	4.1 (2.6–5.3)	0.345
Heart rate (per minute)	177 ± 24a	180 ± 22a	191 ± 18b	0.042
Respiratory rate (per minute)	28 ± 11a	31 ± 14a	47 ± 13b	0.0031
Systolic blood pressure (mmHg)	126 ± 19a	133 ± 9a	116 ± 31b	0.018
LA maximum diameter (mm)	14.2 (12.6–15.8)a	16.0 (13.3–19.1)b	19.6 (18.0–29.9)c	<0.0001
LAA velocity maximum (m/s)	0.51 (0.27–0.88)a	0.44 (0.09–0.62)b	0.13 (0.02–0.45)c	<0.0001
LA:Ao ratio	1.26 (1.07–1.44)a	1.66 (1.5–1.96)b	1.89 (1.79–2.31)c	<0.0001
LA FS (%)	22.5 ± 2.2a	17.6 ± 1.1b	12.8 ± 1.8c	<0.0001
IVSd (mm)	4.29 ±0.51a	7.11 ±0.64b	7.88 ±0.77c	<0.0001
LVPWd (mm)	4.17 ± 0.42a	7.09 ± 0.53b	7.93 ± 0.76c	<0.0001
LV FS (%)	50.3 ± 6.2a	49.0 ± 4.6a	45.7 ± 5.4b	0.004
NLR	1.80 (0.41-8.85)a	2.48 (0.73-7.35)a	5.11 (1.55-48.18)b	<0.0001

*HCM, hypertrophic cardiomyopathy; CHF, congestive heart failure; LA, left atrium; LAA, left atrial appendage; LA:Ao, left atrium to aorta ratio; LA FS, left atrial fractional shortening; IVDd, interventricular septum in diastole; LVPWd, left ventricular posterior free-wall in diastole; LV FS, left ventricular fractional shortening; NLR, neutrophil to lymphocyte ratio*.

*The letters a, b, and c are used to denote significant differences between group. Normally distributed data are represented as mean ± SD, and no nonnormally distributed data are represented as median (range)*.

A total of 43/96 (44.8%) cats died by the end of the study period. One healthy cat was euthanized because of septic peritonitis. Four Stage B cats with HCM were euthanized because of advanced kidney disease (3) and a soft tissue sarcoma (1). All Stage C cats were euthanized because of refractory pulmonary edema (17), refractory pleural effusion (5), arterial thromboembolism (4), or died suddenly (3). None of the cats were lost to follow-up.

**Figure 1:**
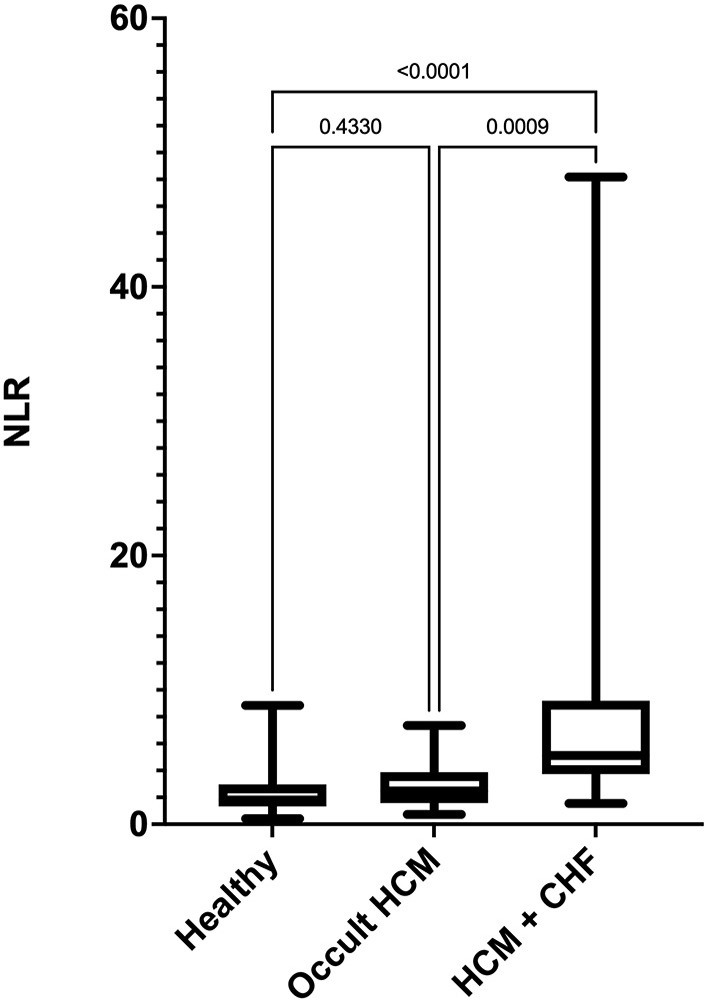
Box-and-whiskers plot of (NLR) in 96 cats. For each plot, the box represents the interquartile range, the horizontal line in each box represents the median, and the whiskers denote the range. The Stage C HCM (*n* = 29) group had significantly high NLR than Stage B HCM (*n* = 29) and healthy groups (*n* = 38). NLR, neutrophil-to-lymphocyte ratio; HCM, hypertrophic cardiomyopathy; CHF, congestive heart failure.

Stage C cats had significantly high NLR compared with clinically healthy and Stage B cats (Figure 1). The NLR was significantly correlated with LAA (ρ_s_ = −0.85), LA FS (ρ_s_ = −0.74), LA_max_ (ρ_s_ = 0.64), LA:Ao (ρ_s_ = 0.61), LVFWd (ρ_s_ = 0.37), and IVSd (ρ_s_ = 0.26). The degree of multicollinearity assessed by the variance inflation factor for each of these significant correlations was <10 (1.74–7.80).

There were significant interactions among echocardiographic variables that were considered when performing multivariable Cox proportional hazard analysis. Significant correlation was present between LA_max_ and LAA (ρ_s_ = −0.62), LA FS (ρ_s_ = −0.81), LA:Ao (ρ_s_ = 0.80), IVSd (ρ_s_ = 0.40), LVFWd (ρ_s_ = 0.56), and LV FS (ρ_s_ = −0.36). Additionally, LVFWd was correlated with LAA (ρ_s_ = −0.42), LA FS (ρ_s_ = −0.54), LA:Ao (ρ_s_ = 0.52), and IVSd (ρ_s_ = 0.34) and LA FS with LV FS (ρ_s_ = 0.42). Significantly higher NLR (*P* = <0.0001) was present in cats with left atrial SEC or thrombus (8.62, CI 4.17−48.18) compared to cats with HCM that did not have SEC or thrombus (2.1, CI 0.79 – 6.83).

**Figure 2:**
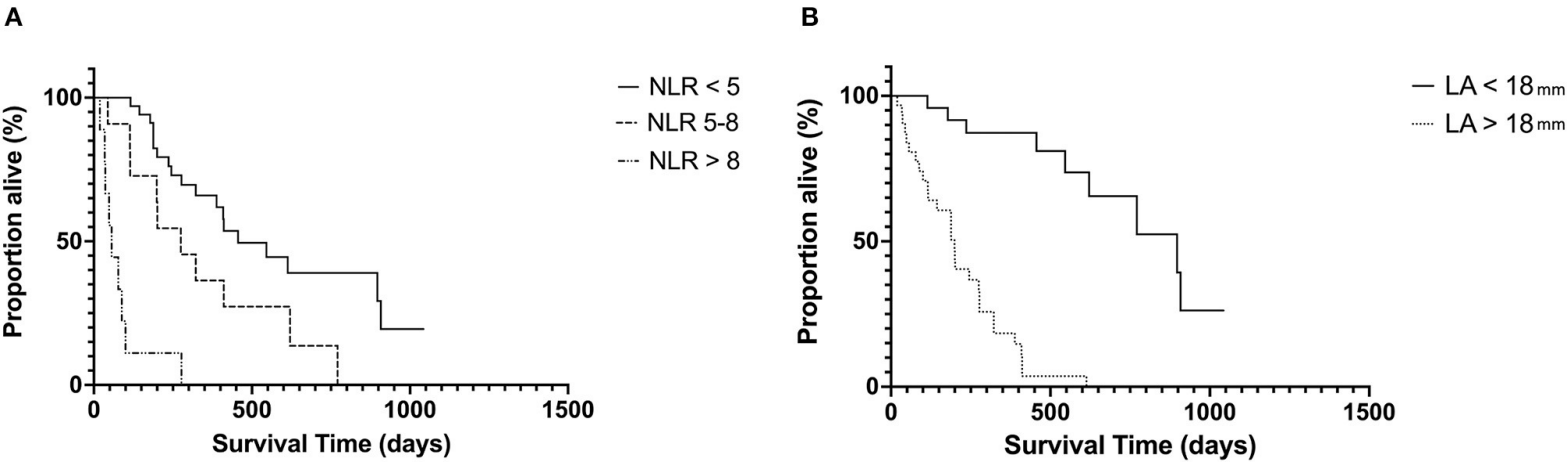
Kaplan–Meier curves showing the effect of NLR **(A)** and LA size **(B)** on survival in cats with hypertrophic cardiomyopathy at the univariable level. NLR, neutrophil-to-lymphocyte ratio; LA, left atrium.

The median follow-up time for the whole population was 344 days (19–1, 500 days). Given a lower than 50% death rate in the whole population, survival analysis was evaluated in only the HCM affected cats. In this cohort, the median survival time (MST) was 265 days (19–1, 043 days). Stage C cats had significantly shorter MST 188 days (19–546) compared with Stage B cats 456 days (67–1, 043 days) *p* = 0.004. Both LA_max_ and NLR had a significant impact on MST (Figure 2). Cats with LA_max_ ≥ 18 mm had a significantly shorter MST (199 days, 95% CI 115–277 vs. 897 days, 95% CI 546–908 days, *p* = <0.0001), HR 9.5, 95% CI 4.59–19.67. There was a significant reduction in MST in the top 2 NLR tertiles (*p* = <0.001). The MST for cats in tertile 1 (NLR <5) was 456 days, 95% CI 322–908 days; tertile 2 was 275 days, 95% CI 114–621 days, HR 2.18, 95% CI.0.96–4.94; and tertile 3 was 56 days, 95% CI 19–100 days, HR 10.26, 95% CI 1.84–57.14.

For all cats with HCM, the follow variables were significant in the univariate Cox proportional hazards model: NLR (HR 1.23, 95% CI 1.11–1.37, *p* = <0.001), LA_max_ (HR 1.78, 95% CI 1.49–2.11, *p* = 0.001), IVSd (HR 2.14, 95% CI 1.38 – 3.32, *p* = <0.001), LVFWd (HR 1.97, 95% CI 1.30 – 2.98, *p* = 0.003), and LV FS (HR 0.89, 95% CI 0.83–0.96, *p* = 0.001). In the multivariable Cox proportional hazards model only LA_max_ (HR 1.54, 95% CI 1.25–1.92, *p* = 0.001) and NLR (HR 1.11, 95% CI 1.03–1.21, *p* = 0.006) remained significant. Indicating that LA size remains a strong predictor of outcome in cats with HCM and NLR may also provide prognostic information. Results of receiver operating characteristic indicated that NLR (AUC = 0.86, 95% CI = 0.77–0.96) provided very good diagnostic accuracy to predict cardiac death in cats with HCM. Using a cutoff for NLR of >3.43 (Youden's Index = 67) provided a sensitivity of 82% and specificity of 86%, and a cutoff of >4.46 (Youden's Index = 53) provided a sensitivity of 58% and specificity of 95% at predicting cardiac death.

When evaluating the Stage C cats alone, the significant variables in univariate Cox proportional hazards model were LA_max_ (HR 1.39, 95% CI 1.09–1.77, *p* = 0.01) and NLR (HR 1.12, 95% CI 1.05–1.21, *p* = 0.001), and in the multivariable analysis only NLR (HR 1.11, 95% CI 1.03–1.19, *p* = 0.002) remained significant, indicating that in this population of heart failure cats, NLR was an independent predictor of cardiac death.

## Discussion

We demonstrated that NLR is a significant prognostic indicator in cats with HCM. Moreover, an increased NLR was associated with reduced MST in both Stage B and Stage C cats suggesting that higher NLR is associated with reduced time to cardiac-related death. Based on Cox proportional models, for each unit increase in NLR, there was an 11% increased risk of cardiac death in cats with Stage B and Stage C HCM. A NLR >4.46 was associated with a 95% specificity in predicting cardiac death.

The NLR is the number of neutrophils divided by the number of lymphocytes. In cats, increases in NLR have been documented in inflammatory and neoplastic disorders (17–20). However, endogenous cortisol and catecholamines will increase under physiologic stress and have the effect of increasing the number of neutrophils, while decreasing the number of lymphocytes. Thus, NLR is not solely an indication of neoplasia or inflammation, and in humans any cause of physiologic stress may increase the NLR (29).

While it is unclear if the elevated NLR in this population of cats was the result of physiologic stress, inflammation, or both, systemic and myocardial inflammation has been documented in feline HCM. Cats with congestive heart failure caused by cardiomyopathies including HCM have demonstrated increased plasma concentrations of tumor necrosis factor alpha (TNF-α) and serum amyloid A concentrations (30, 31). There is evidence to support that TNF-α may be a driving force in peripheral lymphocyte apoptosis further contributing to lymphopenia (32). Inflammatory cell infiltrates, specifically lymphocytes and neutrophils, have been identified in the myocardium of cats with mild preclinical HCM (33). Recently, it was confirmed that systemic diseases with a generalized inflammatory response activate the transcription of inflammatory cytokines and remodeling enzymes in the myocardium (34). While inflammation may be present, it is unclear if this ultimately leads to myocyte injury and death. A specific marker of myocyte injury and death, cTnI, has been shown to predict cardiac mortality in cats and perhaps the combination of cTnI and NLR will provide superior prognostic information than either alone (10). In humans, accumulating evidence has suggested the existence of low-grade systemic and local inflammation in HCM. Mild chronic inflammatory cell infiltration was observed in the myocardium of patients with HCM (35–37). Several studies also reported increased circulating inflammatory markers in HCM such as TNF-α and interleukin-6 (38–40). While associations are continuing to mount, the exact mechanism for the inflammatory cell infiltration of the myocardium in cats and humans with HCM is unclear. Possible etiologies including altered mechanical stress inducing inflammatory cytokine expression (41), hypoxia. and ischemia induced TNF-α, IL-8, and monocyte chemoattractant peptide (42) and triggered inflammatory response during wound repair (43).

One complication of HCM is arterial thromboembolism (ATE) (3, 5, 44). Affected cats often present with left atrial enlargement, impaired atrial function, and reduced blood flow velocities (45, 46). However, not all cats with left atrial enlargement develop an ATE, suggesting that additional factors might contribute to ATE formation and hypercoagulability is suggested in some cases (47, 48). One study demonstrated that the feline myocardium constitutively transcribes proinflammatory and remodeling markers, generally at higher levels in atria than in the ventricular myocardium (49), and specific marker activation in the atria could therefore contribute to an environment that supports thrombus formation. In this population of cats, increased NLR was significantly associated with the presence of SEC or thrombus within the left atrium further supporting an association between ATE and inflammation.

This study has several limitations. This was an observational study and there may have been residual or unmeasured confounding factors as a possible alternative explanation of our observational results. We did not measure variables, including NLR, overtime that could have changed the results. Serially monitoring may improve the prognostic ability of NLR or could identify those patients at greatest risk for progression. This study did not evaluate circulating inflammatory cytokines or antemortem assessment of myocardial inflammation *via* cardiac magnetic resonance imaging, which could greatly improve the understanding of the role of inflammation in HCM. Finally, we did not analyze all-cause mortality and focused solely on cardiac death. It is therefore possible that some causes of death may have been misclassified and given the option for euthanasia; MST may not be interchangeable with different populations and owners.

## Conclusions

Increased NLR is a negative prognostic indicator in cats with HCM and is associated with an increased likelihood of spontaneous echo contrast or thrombus formation in the left atrium and a shorter median time to cardiac death.

## Data Availability Statement

The datasets presented in this study can be found in online repositories. The names of the repository/repositories and accession number(s) can be found in the article/Supplementary Material.

## Ethics Statement

The animal study was reviewed and approved by Institutional Animal Care and Use Committee at the University of Illinois at Urban-Champaign. Written informed consent was obtained from the owners for the participation of their animals in this study.

## Author Contributions

RF: study design, data acquisition and interpretation, and preparation of manuscript SK: study design, data acquisition and interpretation, and manuscript revision. JS, LK, and GW: study design, data acquisition, manuscript revision. All authors contributed to the article and approved the submitted version.

## Funding

This work was supported by Winn Feline Foundation Grants (MTW17-009 and W18-031).

## Conflict of Interest

The authors declare that the research was conducted in the absence of any commercial or financial relationships that could be construed as a potential conflict of interest.

## Publisher's Note

All claims expressed in this article are solely those of the authors and do not necessarily represent those of their affiliated organizations, or those of the publisher, the editors and the reviewers. Any product that may be evaluated in this article, or claim that may be made by its manufacturer, is not guaranteed or endorsed by the publisher.
